# Using Psychologically Informed Community-Based Participatory Research to Create Culturally Relevant Informal STEM Experiences

**DOI:** 10.3390/bs15091249

**Published:** 2025-09-13

**Authors:** Jennifer LaCosse, E. Shirl Donaldson, Thiago Ferreira, Mihai Burzo

**Affiliations:** 1 Psychology Department, University of Michigan Flint, 327 E Kearsley St, Flint, MI 48503, USA; 2 College of Innovation & Technology, University of Michigan Flint, Flint, MI 48502, USA

**Keywords:** CBPR, STEM education, ISE, informal science, co-creation

## Abstract

Systemic racism, high turnovers of teachers and administrators, and deindustrialization in Flint, Michigan, have created an environment that limits the opportunities of Flint youth to engage in and succeed in STEM. This paper describes a partnership between university researchers and Flint community members formed to start the task of addressing this issue. We took a community-based participatory research (CBPR) approach in which we treated community members as co-creators of informal science experiences (ISEs) that take place outside of the classroom. We also integrated psychological research into our research practices and design. To provide context for our work, we review the current literature on ISE and CBPR. We then share our general approach to forming an understanding of minoritized youths’ experiences in STEM in Flint. Next, we discuss how our relationship with the community started, what is working well, the challenges we face, and our recommendations for future researchers. Finally, we discuss the implications of what we have learned and directions for future research.

## 1. Introduction

For decades, rust belt cities like Pittsburgh, Baltimore, Cleveland, Detroit, and Flint were American centers of innovation that provided people with jobs of the future in fields such as science, technology, engineering, and math (STEM). Flint, Michigan, also known as “Vehicle City,” housed some of the first auto-assembly plants in the country and is still General Motors’ longest running assembly plant and a major employer in the area. Several STEM-related job opportunities in Flint have seen 20% growth since 2019, and, out of the top six highest-paying industries in Flint, four require a STEM education (i.e., architecture and engineering; healthcare practitioners; computer and mathematical; and life, physical, and social science; Flint & Genesee Economic Alliance, 2025).

However, since the late 1960s, Flint, like other rust belt cities, has experienced disinvestment and urban decay that has disproportionately impacted Black people (Hightower, 2015). One consequence of disinvestment is the unequal educational opportunities that continues to plague predominantly Black cities like Flint (M. J. Thompson, 2012). White flight and widespread racial segregation in neighborhoods and schools between 1960 and 2010 drastically decreased the tax base used to fund education in Flint (Hightower, 2015), and racial segregation in schools continues to be a problem in the city (United States Government Accountability Office, 2022). According to the U.S. Census Bureau (U.S. Census Bureau, 2023), 56.3% of Flint residents are Black, and 33.3% are living in poverty, which is twice as high as the poverty rates across both Michigan and the U.S. This is problematic because high-poverty schools that are predominately Black are less likely to offer the essential math and science courses needed to prepare students for college and successful careers (United States GAO, 2016). Indeed, within the Flint school district only two of thirteen schools offer calculus, only three offer physics, and none of the schools have gifted and talented education programs (Office for Civil Rights, 2017). Notably, the Flint public school system receives supplemental funds from the state and federal sources to address deficiencies in education. Remediation has been attempted in after-school programs as the population is declining and faculty experience high turnover rates. Yet no substantial gains have been made and the damage from divestment has not been remediated.

As noted above, Flint has also seen high turnover among school administrators and teachers leading to teacher shortages (J. Chambers, 2018; MCEPI, 2024d). Resources and better-prepared teachers flow to wealthier school districts (MCEPI, 2024e) and underpaid teachers with as many as 30 students per class must meet the needs of children in a city where enrichment opportunities—particularly STEM-focused opportunities—are far and few between. The impact of a lack of teachers, resources, and enrichment opportunities on Black and Hispanic/Latinx children in Flint is devastating.

**Figure 1:**
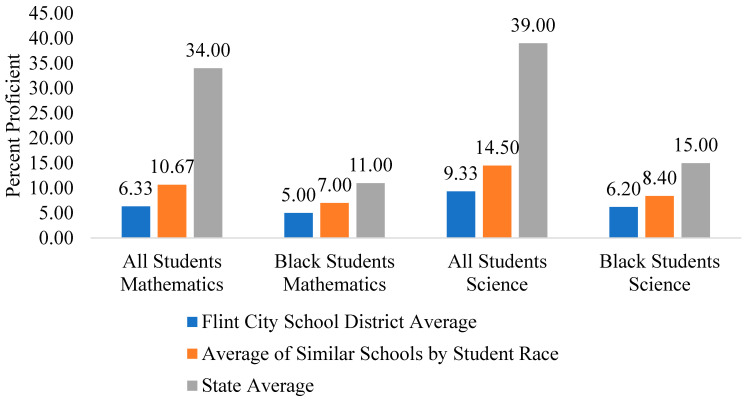
Percentage of students who scored proficient on 2022–2023 state tests by student race. Note: Proficiency scores are based on MSTEP results. Data provided by the Center for Educational Performance and Information (MCEPI, 2024a).

Indeed, Flint children have not kept pace with children in similar school districts across the state even when accounting for race (see Figure 1). Statewide data also illustrate the trends of learning loss in 2023–2024 across high, middle, and elementary school levels (see Table 1; MCEPI, 2024a, 2024c). In both mathematics and science, the percentage of students in Flint who are proficient in the subject is lower than the average percent of students from other schools in Michigan that are predominantly Black and much lower than the state average. Moreover, when the focus is put on Black students, the percent of students who are proficient in math and science are 2–5% lower than Flint students across races and 6–9% lower than the state average. It is also worth noting that in 2024 only 33.66% of Flint students graduated from high school (MCEPI, 2024b). Notably, available data that are comparable to the Michigan school data used above suggests this is also the case for other predominantly Black cities affected by systemic racism (Baltimore City Public Schools, 2024; Pittsburhg Public Schools, 2024; Reyes et al., 2023).

Together these statistics highlight just how underserved Flint’s youth are and the role that systemic racism plays in limiting their opportunities to succeed, particularly in STEM. This mirrors other predominantly Black cities and schools where relatively few Black and Latinx students receive the kind of institutional encouragement, educational opportunities, and preparation needed for them to choose STEM as a field of study and profession (Margolis, 2008). Without proficiency in math and science, youth are less prepared to pursue STEM fields and in turn less likely to be accepted into competitive STEM programs at colleges, universities, and trade schools. This means that those youth are missing out on being eligible for higher-paying jobs in a sector that will make up many of the jobs of the future in both Michigan and Flint (Flint & Genesee Economic Alliance, 2025; Michigan Department of Technology, Management, & Budget, 2025). These statistics also highlight that the STEM opportunity landscape for Flint youth has been ravaged. An opportunity landscape is shaped by time, place, and geographic area, and it impacts people who operate within it (Pinkard et al., 2025). One goal of the current work is to better understand the STEM opportunity landscape of Flint and how to rebuild it through equitable relationships with community members.

## 2. Our Project

In the last two years we have teamed up with Flint community members to begin co-creating culturally relevant and responsive informal science experiences (ISEs). In this paper we wish to share our experiences thus far in the hope that we can help other researchers who seek to address the minoritization of youth in STEM by performing community-based research in cities like Flint. This paper offers recommendations for methodology, but before discussing those recommendations it is important to highlight the components of our project and how they informed our methodology. One key component of our project is our use of psychological literature to inform our approach to doing research and what outcomes we hope to achieve. As we’ll discuss below, there is a plethora of psychological research on minoritized youth in STEM, the utility of ISE, cultural relevance, and community-based participatory research (CBPR); however, much less literature exists on the combination of these four domains.

To set up this important contribution of the present work, we first review the current literature on the psychological experiences of minoritized youth in STEM. Next, we examine the literature on community-based participatory research (CBPR), ISE, and cultural relevance and responsiveness. We then share our general approach to forming an understanding of minoritization in STEM in Flint. Following this, we will discuss how our relationship with the community started, what is working well, the challenges we face, and the key outcomes of our work—including our recommendations for future researchers. Finally, we discuss the implications of what we have learned and directions for future research.

## 3. The Psychological Experiences of Minoritized Youth in STEM

Some of the best predictors of STEM achievement among Black youth are psychological in nature. In a review of research on Black youth (K-12) and STEM, London et al. (2021) found that factors like racism, classism, youth’s perceptions, and relationships with teachers and family predict STEM aspirations and choices. Flint youth have lived through the Flint water crisis and its aftermath, making racism and classism a prevalent part of their lives. Also prevalent is a lack of STEM role models, mentors, and tutors. Role models, mentors, and tutors can buffer against the negative effects of racism, connect youth to STEM, and imagine a future where someone like them succeeds (Bonous-Hammarth, 2000; Lawner et al., 2019; Prins et al., 2010; Steele et al., 2002). As noted, parents can also connect youth to STEM and positively affect their STEM motivation (Šimunović & Babarović, 2020; Starr et al., 2022). Taken together, this research suggests that considering asset-based psychological predictors of Black youths’ pursuit of and achievement in STEM is crucial.

Other important psychological factors associated with persistence in STEM include math and science identification (Archer et al., 2010; Mau & Li, 2018; Tan & Calabrese Barton, 2018), beliefs that intelligence is changeable (Kricorian et al., 2020; Seo et al., 2019), and perhaps most importantly, belonging (McMahon & Wernsman, 2009; Walton & Cohen, 2011). Greater feelings of belonging are associated with greater STEM identification, interest, persistence, and performance among minoritized students (Kricorian et al., 2020; Xu & Lastrapes, 2021). Increasing feelings of belonging can eliminate racial and gender disparities in performance and persistence in both STEM (Abrica et al., 2022; LaCosse et al., 2020) and non-STEM contexts (Walton & Cohen, 2011). As we elaborate below, we hoped to increase Flint youths’ feelings of belonging in STEM in part by including community groups they are already a part of—specifically churches—in the design and implementation of our intended program of research.

## 4. Community-Based Participatory Research

**Figure 2:**
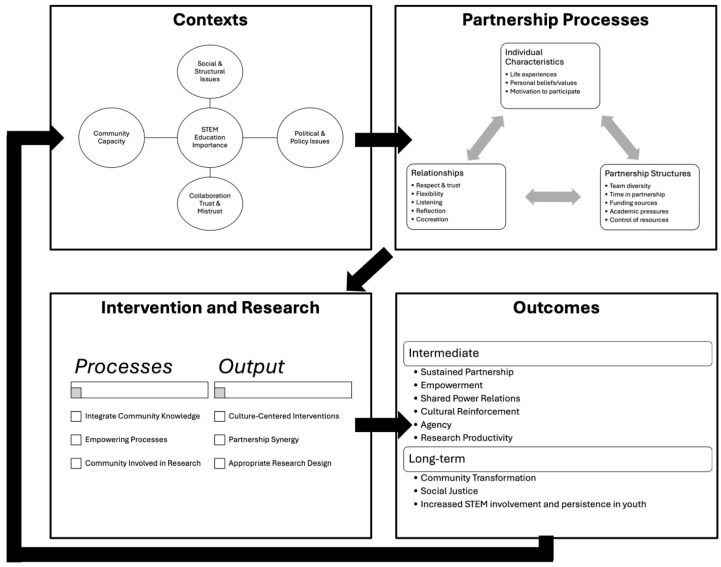
Conceptual CBPR model. Note: Adapted from amoshealth.org (2017).

Community-based participatory research offers researchers an invaluable opportunity to understand the lived experiences of racial and ethnic minorities (Breland-Noble et al., 2024). CBPR involves the formation of meaningful and equitable partnerships with communities to tackle research questions relevant to the needs of the community (Scher et al., 2023). For an overview of the complexity of CBPR see Figure 2. As can be seen in this figure, CBPR research extends beyond the goals of the researchers and their preconceived notions of what is taking place in the community. Instead, it directly assesses the needs of communities and seeks to address those needs in a manner that benefits the specific community researchers are interested in.

Notably, among racial and ethnic minorities, CBPR has been used to increase participation in clinical trials (for a review see McFarlane et al., 2022), improve food security (for a review see Doustmohammadian et al., 2022), address public health problems (see Belone et al., 2016; Hailemariam et al., 2020; Key et al., 2019 for research in Flint), and formulate sexual health interventions (McCuistian et al., 2023). Relevant to our focus on systemic racism’s role in creating underrepresentation in STEM, a meta-review by Ortiz et al. (2020) demonstrates that CBPR is also associated with organizational changes, cultural revitalization, and community transformation as seen in Figure 2.

While there is a history of CBPR in STEM education (e.g., Beltrán-Grimm, 2025; Bermudez et al., 2023; Pesch et al., 2022; Rumala et al., 2011), there is a lack of published research that co-creates with local community members (e.g., church leaders, community organizers, and prominent influential individuals) rather than parents, youth, or local non-profits. Some research has focused on community-based learning in which community needs are considered, but community members were not treated as equal partners when it came to the design and implementation of STEM programs aimed at decreasing minoritization in STEM (Calabrese Barton & Tan, 2018; Kennedy et al., 2016; Nation & Hansen, 2021). Other STEM education research among minoritized populations does recognize the complex relationships between researchers and community stakeholders; however, some of this research has been performed outside of the United States (Delaine, 2021; Delaine et al., 2018; Sadler et al., 2018), reviews relationships with formal advocacy groups rather than local community members (C. A. G. Mwangi et al., 2023; J. D. Thompson & Jesiek, 2017), or focuses on relationships with parents (Beltrán-Grimm, 2025; Bermudez et al., 2023; Salazar et al., 2025). The present work makes a unique contribution to this body of literature by specifically focusing on our academic research team’s experiences forming equitable relationships with *community members* in a city marred by systemic racism in the United States.

The present work also contributes to the literature on CBPR in the domain of psychology (for reviews see Levac et al., 2019; Rodriguez Espinosa & Verney, 2021). Rodriguez Espinosa and Verney (2021) systematically reviewed CBPR research in psychology and found that, out of the 311 articles found, the majority focused on physical health (*n* = 93), mental and behavioral health (*n* = 63), or theory and methods (*n* = 66). In contrast, only seven of the articles found were related to education and curriculum development. Our own review of psychological CBPR research in the domain of STEM education revealed that much of this work uses undergraduate or high school students as co-researchers (e.g., Guy & Feldman, 2023; Jacquez et al., 2020; Siciliano et al., 2018) rather than community members as co-creators of STEM programs for minoritized youth, which is the focus of our work.

## 5. Informal Science Experiences

To mitigate persistent inequality in formal science education, many communities have endeavored to expand STEM education to include informal science experiences (ISE; for a review see Alexandre et al., 2022). ISEs take place outside of the formal K-12 education system. Relevant to our work, ISEs include summer camps, watching presentations by scientists, completing STEM activities (e.g., robot building, 3D-printing, app development, observing nature, etc.), receiving mentorship, being tutored, attending after-school events, and taking trips to places like museums, science centers, botanical gardens, and zoos (Acosta et al., 2021; Allen et al., 2019; Ghadiri Khanaposhtani et al., 2018; Plummer & Small, 2018; Young et al., 2019). Past research indicates that ISEs such as these are positively associated with STEM literacy, interest, and participation (Alexandre et al., 2022; Young et al., 2019; Young et al., 2016). There is also initial—primarily qualitative—evidence that ISEs can be leveraged to support STEM identity development and participation among Black and Hispanic/Latinx children (Godec et al., 2022; Kennedy et al., 2016; Kuchynka et al., 2022). For example, STEM summer camp participation has been linked to creativity in STEM (Bicer et al., 2017), learning motivation (Binns et al., 2016), STEM interest (Ghadiri Khanaposhtani et al., 2018; Kong et al., 2014), forming personal connections with STEM (Anand & Dogan, 2021), and positive perceptions of STEM (Binns et al., 2016; Prins et al., 2010; Vela et al., 2020) across multiple age ranges.

Despite the ability of ISEs to help minoritized children in STEM, it is important to recognize that there are barriers in access to some types of informal learning experiences. As noted above, one of the more common types of ISEs are visits to places like museums, science centers, botanic gardens, and zoos. Although these are great experiences, on their own, they may not be enough to engender STEM interest among Black and Hispanic/Latinx youth. These types of visits are not representative of the society as a whole, but favor the participation of predominantly White, middle-class suburban/urban populations (Bevan et al., 2020; DeWitt & Archer, 2017).

In Flint, the YMCA and Boys and Girls Club in the Flint area have attempted to create learning ecosystems that offer some STEM activities in the form of after school programs. However, there is no evidence of these programs making an impact if standardized test scores or graduation rates are used as metrics. In addition, the Sloan Museum (science exhibits and auto history collection), the Longway Planetarium, the Whiting Auditorium (music performance), and the Flint Institute of the Arts all possess nationally important collections and learning opportunities. These kinds of non-school resources have always been a part of the fabric of the city. But as Falk and Dierking (2010) acknowledged, longitudinal studies of urban and suburban children in metro areas like Baltimore demonstrated that standardized academic performance always dipped for “urban students” over the summer months. Similarly in Flint, despite the wealth of hands-on science or arts institutions, the informal knowledge and practices were not always presented in a way that moved beyond elite epistemologies and practice. Some notable recent programming and exhibitions have moved beyond the western European canon. Nevertheless, located near downtown, in the so-called “cultural campus” neighborhood, these brick-and-mortar institutions have not always been seen as welcoming by Black or Hispanic/Latinx youth in the city.

## 6. Cultural Relevance and Responsiveness

To be effective, ISEs (as with formal learning experiences) must be rigorous, affirming, validating, conversation rich, and authentically engaging, with real-world problems and issues connected to children’s lived experiences (for a review see B. A. Brown, 2021). That is, informal learning, especially for minoritized youth, should be both culturally relevant and culturally responsive (for a review see Ladson-Billings, 2021a; see also Leonard et al., 2019; Scott & White, 2013; Stevens et al., 2016). Both cultural relevance and cultural responsiveness are asset-based approaches to teaching, which recognizes and validates youth’s pre-existing talents, skills, interests, concerns, and values (Celedón-Pattichis et al., 2018; Harper, 2010; Montañez, 2023)—paralleling our focus on asset-based psychological constructs. These approaches are important because racial minority youth are equally interested in STEM as their White counterparts, but they are less likely to participate in ISE because the benefits of participating are not always made obvious to them (Godec et al., 2022), or they are not provided with the connection with their everyday lives (Christidou et al., 2021; Nicolaisen et al., 2023; Vela et al., 2020). In line with the work of Ladson-Billings (1995, 2021a, 2021b, 2022), our goal was to provide this connection and empower students to develop critical consciousness and challenge the status quo.

Culturally responsive ISE emphasize using students’ cultural knowledge and experiences to make learning more effective (Ladson-Billings, 1995). For example, youth who used personalized heat maps to provide a visual representation of basketball players’ strengths and weaknesses showed a significant increase in STEM interest after the activity (Drazan et al., 2017). Black and Latinx girls’ sustained engagement in a COMPUGIRLS program was predicted by their use of coding to express themselves and help their communities (Scott & White, 2013). Moreover, Black youth in afterschool programs who learned how to program robots and develop digital code for games that were personally meaningful reported gains in self-efficacy (Newton et al., 2020). This research suggests that developing culturally relevant ISE with community members via a CBPR approach could be an effective means of promoting positive psychological outcomes associated with STEM interest, persistence, and performance.

The little psychological CBPR research in the domain of STEM education in a minoritized context we were able to find where community members were treated as co-creators is informative. Navickis-Brasch et al. (2014) developed a community partnership with Tribal Communities to develop a STEM summer camp that positively affected youth’s attitudes toward STEM. Despite these promising results, Navickis-Brasch et al. (2014) identified several lessons learned that are relevant to our work. First, community members involved in the project did not feel like equal partners, which led to less community participation. Community members specifically mentioned a lack of communication. Parents wanted more information to be provided both before the summer camp and during the summer camp and guest speakers at the camp indicated that it was unclear what they were being asked to do. Navickis-Brasch et al. (2014) also suggested that community meetings be held both in person and online and highlighted the need to co-develop curriculum rather than presenting a curriculum for community feedback. Finally, and perhaps most importantly, community members expressed concerns that researchers were prioritizing publishing youth’s data over using it to improve youth’s experiences. These are valuable lessons learned that we return to when discussing our approach.

The history and context of the city of Flint—which we discussed previously and return to shortly—offers another lesson learned that is relevant to culture-specific teaching practices. As culture-specific teaching practices evolve, they engender the development of culturally sustaining and anti-racist pedagogies that work to undermine systemic racism (Ladson-Billings, 2021a). STEM experiences are shaped by other sociopolitical factors such as corporatism and consumerism (Vakil & Ayers, 2019), which are both major issues in the city of Flint. Given these findings, cultural relevance and taking an asset-based approach have been central tenets of our work.

## 7. Our Journey

**Figure 3:**
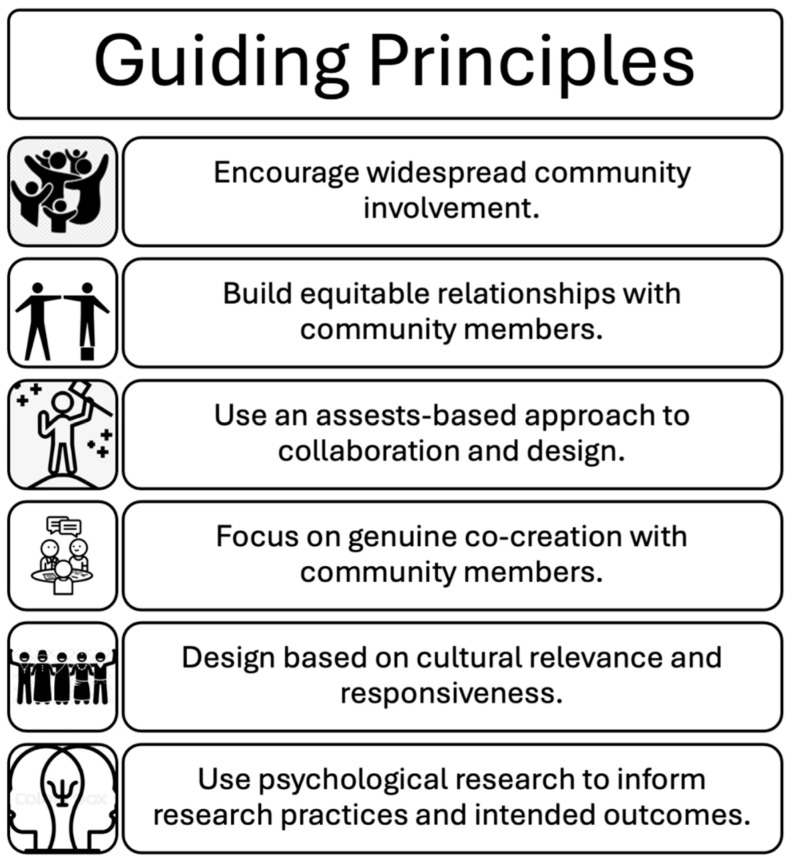
Our guiding principles.

To share our journey, we will start by talking about the context of our work in Flint, Michigan. Next, we introduce our team—both researchers and community members. Following this, we provide an overview of our journey including the conceptualization of the project, our first meeting with community members, our original goals, and how things progressed. We then discuss what is working well and the challenges we have faced. Throughout our discussion, when possible, we include quotations from community members who attended our team meetings—some but not all meetings were recorded. When not possible, we relied on the meeting notes researchers took. For an overview of our guiding principles see Figure 3.

### 7.1. The Context

Thus far we have summarized the educational landscape in Flint. To fully understand our community and our relationships with community members it is important to discuss the role of trust. Both historical and contemporary systemic racism in Flint have led to distrust in the city’s institutions, government, and potential investors (Hightower, 2015). The Flint Water Crisis deepened this distrust because the local government was responsible for the crisis—by failing to make the water infrastructure safe after being told of the consequences—and denied that the water supply was tainted for a long time (Morckel & Terzano, 2018). Flint residents are still experiencing negative health outcomes as a result of consuming the tainted water, and, as of 2022, 5% of the city has still not had the lead pipes that caused the crisis replaced (D. Brown, 2024; Department of Environment, Great Lakes, and Energy, 2022). Moreover, exploitation by researchers studying the effects of the crisis has made Flint residents skeptical of researchers’ intentions (Carrera et al., 2019). This is problematic for our own work because community trust is an essential component of CBPR (Lucero et al., 2020; Moore de Peralta et al., 2020).

### 7.2. Our Team

Past CBPR research indicates that diverse teams, in terms of both community members and researchers, have many benefits (e.g., Chandanabhumma et al., 2023; Muhammad et al., 2015; Wallerstein et al., 2008). It can help address power differentials (Muhammad et al., 2015), foster mutual learning (Wallerstein et al., 2008), and foster shared understanding (McFarlane et al., 2022). Perceived diversity also increases participation in decision-making (Chandanabhumma et al., 2023). In addition, diversity in researchers’ disciplines facilitates effective CBPR (Bowker, 2021; Hohl et al., 2022).

#### 7.2.1. University Team Members

The first author is an Assistant Professor of Psychology who studies antiracism and minoritization in STEM. She is a White woman from a working-class family and is a first-generation college student. The second author is an Assistant Professor of Industrial Technology who previously worked for a top-tier automotive supplier owned by her family. As a project manager and scholar, she studies underrepresented minorities in STEM and entrepreneurship. Academia is her second career; hence she brings a wealth of experience in relationship building, mentorship, and networking. Community-engaged research was a natural evolution as she transitioned from the business world to academia, bridging the gap for social mobility in underserved populations. The third author is an Assistant Professor of Information Technology and Informatics who studies the use of user preferences, optimization algorithms, and artificial intelligence techniques to address several software engineering problems such as software requirements, software testing, and software refactoring. The fourth author is an immigrant and an Associate Professor of Information Technology and Informatics who studies heat transfer in microelectronics and nanostructures, thermal properties of thin films of new and existing materials, multimodal sensing of human behavior, and computational modeling of forced and natural heat convection. Each one of these professors brings their own area of expertise and research, but more importantly, they are interested in engaging with the community and broadening participation in STEM. This was especially important in the Flint area and underserved populations like the Black community who resided within the immediate area of the campus.

As each professor introduced themselves to the community partners, positionality statements were made in an informal way. Two of the professors were women, and the two males were both immigrants, born outside of the United States. These four individuals embodied atypical stereotypes of American college professors. They shared different lived experiences from many of their colleagues on campus and brought a different perspective to the community.

If performed fittingly, the positionality statement can bridge a gap by finding common ground with community members. Common ground can be established by a shared belief structure, identifying mutual challenges or struggles, similar experiences, and a desire to see positive change. However, the statement is only as powerful as the behavior that accompanies it. Authenticity is a must. Acknowledgement of the power differential can be viewed as part of the system that oppressed underserved communities while hindering academic professionals that seek to serve them. There can be more one power differential at play at once. For instance, while the professor may have been perceived as holding more power than the community members, in this scenario, the National Science Foundation held the power over the professors in the funding decision.

#### 7.2.2. Community Team Members

To reach Flint children not associated with our churches, we also formed partnerships with two youth organizations. Both organizations serve local youth by, among other things, coordinating after-hours STEM learning activities at facilities on the northside, eastside, and southside of Flint. There is some evidence that these types of STEM programs are associated with increased teacher perceptions of children’s STEM skills and identity and a sense among children that STEM has the potential to impact their community (Kennedy et al., 2016; Tan & Calabrese Barton, 2018).

This project started with a focus on local congregations. After identifying congregations, primarily through online searches, the Urban Institute—whose director was also a part of this project—sent out sixty-two letters to faith leaders inviting them to participate. The initial response was enthusiastic but limited in number. As common in certain subgroups in urban overstudied areas, trust was not immediately assumed. Under normal circumstances, some individuals are more trusting than others. Individuals have varying experiences and tolerance levels within any community. However, a community that has been repeatedly neglected, poisoned, and then studied like “laboratory animals” has good reasons to distrust outsiders. Trust must be built with respect and honesty. Researchers must earn the right to enter these spaces and gain acceptance by being transparent and acknowledging the uncomfortable realities. To this end, there was a lengthy acclimatization period as members of the research team exchanged life stories with the community partners from various congregations. The exchange of stories and experiences provided a common ground for discussion of needs for change in the local community, especially with respect to education, career preparation, and systemic barriers to social mobility.

One of the community partners, a college graduate and youth leader, shared her concerns about her son’s career path. Her son was a Black male with a doctorate in computer science but was unable to land a position in his chosen field of study. It became evident that this young man was successful in the classroom but did not receive the professional development required to navigate the job market. The second author of this manuscript leveraged her network to help find professional employment in computer science, by introducing him to potential hiring managers, guiding him through the application process and facilitating a mentoring relationship with an internal manager at the target company. This young man is still employed by that company at the time of this writing. His mother, the community partner from a rather large congregation, will attest to the commitment of this research team to make change and serve others. This validation encouraged other representatives to support the goals of the project with less hesitance.

The team members were invited to events at each of the participating congregations. A few of the team members attended several events to gain a better understanding of the congregational culture. Showing up for these events provided opportunities to converse with youth leadership outside of scheduled research meetings and build more authentic, individual relationships. Members of the research team also invited community partners to various events on campus that may be of interest. This exchange of invitations provided a broader perspective of each organization’s missions and priorities.

It is important to acknowledge that the congregations involved were not monolithic. Most of the congregations were predominantly Black, but a few were multiracial and non-denominational. There was wide representation across the types of congregations involved (Baptist, Methodist, COGIC, etc.); however, they shared a deep concern for the youth in Flint, especially the youth of color and lower socioeconomic status (SES). These partners often commented on the uncertainty of the economic viability in the immediate geographical area and the lack of employment in comparison to previous decades of prosperity. This shared concern fostered cohesion in the team.

### 7.3. How Things Started

#### 7.3.1. Conceptualization

One warm Sunday morning the second author—who conceptualized this project—attended church with her family. It was in mid-June, and the young folks who were graduating from high school were being recognized by the congregation. As often seen in the Black community, education is supported and celebrated. Therefore, when kids graduate from high school, the congregation applauds them and supports them financially with scholarships whenever possible (for an extensive list of available scholarships see Scholarships 360, 2024 among other databases). For centuries, literacy and learning have been core tenets of the church. During Reconstruction and the early decades of the century that followed, schools shared space with Black churches, and Sunday school activists were committed to advancing literacy (Haggler, 2018). Indeed, many HBCUs, such as Wilberforce University, the first college to be founded and operated by Black individuals, were launched by churches (Abelman, 2013). Today, the church is still an engine of social change in Black communities, and it still has a role to play in promoting academic success (McCray et al., 2010; McIntosh & Curry, 2020). Indeed, the Black Church has been the nexus of every meaningful social, economic, and political movement in Black life as documented by major historians, sociologists, and educators (Du Bois & Eaton, 1899; Gates, 2022; Lincoln & Mamiya, 1990; McIntosh & Curry, 2020).

Notably, the description of the second author was part of her positionality statement without labeling it as such. In her introduction to the group, she shared her background in terms of her biological family and family of faith to build common ground. The initial idea of “Vacation Bible School” (VBS) was culturally relevant and made sense to the community members. These life experiences helped her connect to the community. Although this project has yet to be funded or fully implemented, the connection to the community created fruitful conversations and launched relationships for other community-based projects with adjacent teams as in the parents study we discuss below.

Looking at the community-based structure of the Black Church gave rise to the idea of examining how this structure supports ideas and priorities. Life purposes, time, resources, and goal setting is often developed with support from the leadership in houses of worship. For example, a lot of these churches offer VBS, a spiritual but fun type of summer camp for kids. Vacation Bible school typically lasts one week in the summer. People in the congregation volunteer to make it happen for the youth of the congregation every year while kids are out of regular school. They are typically either half a day or sometimes three to four hours in the evening depending on the work schedules of the congregations. During this time, children ages five to twelve years old are engaged in learning Bible verses, songs, and stories. They do arts and crafts, they develop a skit, and they get a snack, play games, and hang out with their friends and family for this week during the summer every year. Many kids go to the one at their church and then, in subsequent weeks, may attend one at their neighbor’s church or an affiliate church, which means they get this experience two or three times over the summer. In addition, throughout the year youth may attend Sunday school before or after they do structured-worship service every week. Importantly, there is evidence that Sunday school participation is associated with academic success (Horwitz, 2022), which mirrors the effects of regularly participating in ISE (Horns et al., 2020).

As researchers, we wondered what would happen if we used the VBS and Sunday school model for STEM education? Could we create an informal setting—being outside of the classroom but formal in structure—by working with the faith-based community? Houses of worship are supported by extended family and friends, and the leadership in the church also places priority on this type of activity, meaning the pastors, deacons, or elders in the church are committed to supporting their youth. Moreover, the congregants tend to support youth outreach and encourage youth participation. So, if we could use this same VBS and Sunday school model to introduce and elevate STEM interest, might we be able to impact youth in a meaningful way? Could we encourage youth to embrace STEM and focus on it when school resumes?

STEM summer camp participation has been linked to creativity in STEM (Bicer et al., 2017), learning motivation (Anand & Dogan, 2021; Kwon, 2017), STEM interest (Chapman et al., 2020; Mohr-Schroeder et al., 2014), forming personal connections with STEM (Ghadiri Khanaposhtani et al., 2018), STEM career interest (Kong et al., 2014; Vela et al., 2020), and positive perceptions of STEM (Üçgül & Altıok, 2022) across multiple age ranges. Each college at the university has successfully conducted STEM camps for several years on a smaller scale, usually involving 20–25 students; however, it was not designed to be culturally relevant and tends to attract more affluent youth with a minimal cost for attendance. Moreover, one of the most recent camps had so few students sign up that the camp was cancelled. This suggests that community members are not aware of the camps and/or are unable to send their child to camp for some reason.

Our conversations with community team members about why more youth attend these camps confirmed this line of thinking. For example, one community member said,

“And so many other things like summer camps. I did them for like several days and I saw the excitement that came out of it. I went to what we call deep programs when we go to high schools in the surrounding areas before you used to get like one, two, students, now (…) when I went, we got like seven, eight, ten. So, (…) I feel that we make an impact whatever we do we just by you know by putting the time and effort into it will solve that (…)”

#### 7.3.2. Project Initiation

To start to answer these questions, the second author reached out to the director of the Office of Research at our university and shared her idea. He thought it was quite novel and would be a good fit for a National Science Foundation Racial Equity in STEM grant that he and the director of the Urban Institute had discussed applying for. Both directors contacted other faculty members that seemed like a good fit, and an initial team was formed. Next, the Urban Institute identified 62 churches, and emails and letters were sent to the leadership of each congregation explaining our idea, purpose, and what we hoped to develop to serve their youth.

The director of the Urban Institute decided that other community organizations such as the Boys and Girls Club along with the Sylvester Broome Empowerment (SBEV) Community center should be invited to meetings and included in the project. She was concerned about youth that were not affiliated with congregations and sought a larger number of participants to make the project more attractive to funding bodies such as the NSF. These organizations were invited to future project development meetings. Although neither of their representatives ever attended a planning meeting over an 18-month period, they quickly submitted sizable budgets to cover parts of their organization’s on-going overhead expenses to be funded by the project with the promises of participation.

Starting in June of 2022, the head pastors, youth pastors, and interested congregants were invited to meetings to discuss the possibilities. In accordance with past research (Tricco et al., 2018), the initial meeting started with ice breaker activities getting to know everyone around the table. Along with usual demographics typically shared in individual introductions, everyone shared why they chose to attend the meeting and gave details on their professional backgrounds. While describing their professional journeys, many shared tales of unfair treatment at school and on the job. They also gave examples of their willingness to persevere and advocate for change. This level of self-disclosure was essential as psychological research demonstrates that self-disclosure promotes liking (Sprecher et al., 2013), feelings of closeness (Sprecher, 2021), and perceptions of the trustworthiness of researchers (Altenmüller et al., 2023).

The first several meetings were attended by seven to twelve community members each month. A few congregants attended out of curiosity. Most were there because the pastor or leadership of their congregation sent them to represent the membership, while others were community leaders interested in helping Flint youth. The attendees were not always the same individuals from each congregation, so there were some reintroductions and briefings necessary to keep everyone abreast of current developments at each meeting. After eight meetings, a Zoom option was added to the in-person meetings for convenience as both university researchers and community members traveled and managed schedule conflicts. Notably, adding the Zoom option was important because we wanted to avoid uneven participation based on social class (e.g., excluding community members without transportation to meetings) or caretaker status (e.g., community members not having alternative care for their children and/or loved ones). This is in line with the work of O’Leary et al. (2019), which highlights that research designs can reproduce racialized exclusions if researchers are not careful.

### 7.4. How the Project Progressed

In less than a year, everyone was on a first-name basis, creating an atmosphere of informality and a willingness on everyone’s part to share their experiences and opinions. Researcher team members tried to relate to community members personally and professionally to reduce power distance in the working environment (Mayan et al., 2016; Ochocka et al., 2002). In addition, structural and systemic racism and critiques of systems of power were frequently discussed, which is in line with recommendations for equity-seeking research (Ahn et al., 2024; Bang & Vossoughi, 2016). The participants came from various congregations throughout the local area. Typically, the congregational representatives were youth pastors, group leaders, and parents selected to be the voice of the youth. These representatives were usually selected by the pastor of each congregation. We, the investigators, did not select the individuals nor were there any guidelines or requirements to be accepted into the project. They each brought their lived experiences along with their concern for the youth. When questioned about the gaps in preparation for future careers, time was taken to ensure that everyone was heard.

During many meetings community members related stories about their experiences with Flint youth. The meetings were held monthly at dinner time at the local library, typically scheduled 6 pm to 7:30 pm in person. If inclement weather became an issue, the meeting would be transitioned to Zoom. The university team provided casual meals such as pizza or sandwiches for the community partners. Mealtime provided an opportunity for casual discussion that led into more formal conversation. The agenda, developed by the principal investigator, was driven by the team’s desire to better understand the partners’ needs and their desire to help the youth in the community. The focus was on STEM education and preparing for jobs of the future in alignment with the goals of a targeted National Science Foundation (NSF) grant.

While some experiences were positive, many more were wrought with a sense of helplessness and true concern for young people. Once concern expressed by a community member was that, if we give youth the skills they need to succeed, we may be able to preserve their connection to the community by encouraging them to stay in Flint but commute:

“I know you know [what] the concern is, OK. We’re going to give some of the kids you know the skills so they can move out of the community, but frankly, I mean in this day and age, a lot of us do commute, right. So, if you have somebody from Flint, (…) [if] they work somewhere like 20–30 miles away. They have the connection with the community.”

After the first meeting with the community partners, the university team reconvened to process the information gathered and determine the strategy for managing future meetings and developing the theoretical constructs for the project. The team started by acknowledging that the community partners had several unmet needs, hence the idea of traditional research was not attractive. The project evolved to community-based and intervention research, needing to be positioned and described in a manner more acceptable to local residents. They had been “studied” enough and sought agency and resources to potentially change the career trajectories for their children and grandchildren. For example, one parent said,

“So, one thing that I see that children struggle with is the influencers that influence the children are mostly people who have things, who have money, and who have jewelry. who have things they’re on the internet, TV, whatever. They’re the influence, so how do I get my children involved in these things? I have to show them a way that they can make money now with technology with STEM. How can that be an actual avenue to show kids (…)”

### 7.5. Funding

As a team we realized that major funding was necessary to make our ideas a reality. Guided by the Director of Research, we decided to submit a grant application for a NSF grant designed to increase the representation of racial minorities in STEM using an antiracist approach. As previously noted, our original idea was to create a STEM summer camp; however, our community team members argued strongly for a frequent series of STEM activities, advocating biweekly—every other week—learning activities at church facilities and youth organization locations. Thus, we added this to our NSF proposal. In addition, based on previous research and community feedback we added the inclusion of formal mentorship, advising, and tutoring. STEM mentors, role models, and tutors play a key role in promoting the participation of underrepresented youth in STEM (Kong et al., 2014; Üçgül & Altıok, 2022; Young et al., 2016). Peer-to-peer STEM mentorship (pairing a youth with an undergraduate) is particularly effective at increasing STEM interest and positive attitudes toward science (P. N. Mwangi et al., 2022; Walan & Gericke, 2021), above and beyond the effects of mentorship from teachers (Horsford, 2010).

Finally, we proposed the measurement of several key psychological constructs that have been shown to positively impact racial minorities’ STEM interest and persistence—rather than focusing on constructs related to racially based deficits. First, a sizable literature indicates that math and science identification is engendered at a young age, and developing a STEM identity early on is essential to positive STEM outcomes (Archer et al., 2010; Mau & Li, 2018; Tan & Calabrese Barton, 2018). Moreover, research suggests that ISE and mentor experiences engender science identification (Hernandez-Matias et al., 2020; Kuchynka et al., 2022), as does a connection between cultural values and science (K. H. Collins, 2018). Another critical factor we wanted to examine is feelings of belonging in STEM—which as previously discussed is key to success in STEM—given initial evidence that participation in ISEs is associated with increased feelings of belonging (Hoffman et al., 2021; Stanich et al., 2018).

We also proposed assessing STEM self-efficacy, which is also positively associated with science identification, belonging, STEM interest, and STEM performance among underrepresented racial minorities (Mau & Li, 2018). Importantly, ISEs have been shown to increase STEM self-efficacy (Hoffman et al., 2021; Newton et al., 2020). Related to increased STEM belonging, STEM self-efficacy, and STEM performance among racial minorities are growth mindsets—beliefs that STEM ability is not fixed and can grow and change (Aronson et al., 2002; Blackwell et al., 2007; Rattan et al., 2012). Like our other constructs, ISEs promote the development of growth mindsets (Cartwright & Hallar, 2018). ISEs also promote self-esteem (Simpson & Parsons, 2009), which is associated with lower drop-out intentions among racial minorities in STEM (Balta-Salvador et al., 2022). Also related to the STEM outcomes discussed above and a frequent moderator of academic outcomes among racial minorities is racial identification (Deemer et al., 2022; Kirby & Kaiser, 2020).

Finally, we proposed assessing cultural capital generally and science capital specifically. Social capital is the advantage people earn through the social resources they have (Eastis, 1998). Social resources, or a lack thereof, can include systemic factors like poverty rates and neighborhood diversity (Yoon, 2021); however, it can also include cultural factors like familial support, being part of a church community, having role models, etc. (Adler & Kwon, 2002). Based on the recommendations of the Institute for The Social Capital Institute (2023), youth will be asked about three dimensions of social and cultural capital (structural, e.g., neighborhood diversity; relational, e.g., support structures like churches; and cognitive, e.g., shared emotional connections). Cultural capital is influenced by both individuals’ surroundings and their understanding of the world (Bourdieu, 1990, 2000). Like social capital, science capital is the collection of science-related attitudes and beliefs that someone can earn that supports and enhances their pre-existing strengths (e.g., attitudes toward science, perceived utility of science, science literacy, familial support of science, and having science role models) (DeWitt & Archer, 2017; Moote et al., 2020). This collection of science-positive beliefs and attitudes are associated with greater ISE involvement (Christidou et al., 2021; Quirke & Hossain, 2023).

### 7.6. Waiting

The next challenge became keeping the group engaged while waiting on a funding decision. The NSF reviews proposals annually, and the turnaround time is typically four to six months. The community partners were informed about the waiting period and the turnaround time. The decision was made to meet less frequently, bimonthly—instead of monthly—and via Zoom rather than in person. To keep these partners engaged, the team visited various congregations to continue building relationships, expand the network, and gain a better understanding of the culture of the individual entities. The congregants invited the professors to church services, anniversary celebrations, concerts, theatrical performances, youth outings, fishing contests, and “back to school” events. Three of the four professors accepted these invitations, which is important because research on the psychology of team building indicates that opportunities for interpersonal exchange reduce reliance on stereotypes and assumptions among team members and promotes team cohesion (Bell & Brown, 2015). During one of these events research team members brought a large poster that listed “STEM Careers Your Child Will Love,” university swag, and a robot. These visits to congregational events sparked an idea for an additional research project with Flint parents that we discuss below, but more importantly they helped create familial relationships with the community and demonstrated our commitment, both of which are key to successful participatory research with Black communities (Harrington et al., 2019).

Relationship management is crucial for co-creation, team building, and successful CBPR (M. Lee et al., 2020; Skipper & Pepler, 2021; Suarez-Balcazar, 2020). Concurrently, the university partners invited community partners to events on campus such as summer camps, guest speaker series, and student engagement opportunities. The team also sponsored workshops to assist families complete financial aid forms. Notable, these university activities were mentioned in previous meetings and social gatherings as being needed by residents.

The focus of these interactions was to maintain a project team while developing a fundable plan for full implementation. Given the scale and magnitude of the intervention community members signified was required to be impactful, a pilot program was not an option. A significantly scaled-down pilot would not garner full participation or produce measurable results.

Unfortunately, no STEM programs were piloted. The original idea was to use the Vacation Bible School model to conduct STEM interventions. However, the community partners wanted a program that was much more comprehensive with many more touch points throughout the year. Broader concepts were formed, but without adequate funding and administrative support, a scaled-down version could not be piloted. The team later learned, after two years of meetings and conversations, that none of the churches were still having Vacation Bible School in the summer.

Ultimately, the proposal was submitted to the NSF, and the waiting for the decision began. During this waiting time, national news reports emerged highlighting the lack of families completing the FASFA. These forms were changed for the upcoming academic year, and there was widespread confusion amongst high schoolers, families, and academic advisors. This became the project to collaborate with the partners. Without funding, the professors, with the support of campus experts, hosted a financial aid, FASFA, workshop to help local families complete the necessary forms to apply for financial aid. This event was well organized and publicized, yet only a few people from the community attended. None of the congregational representatives or their relatives attended this event!

In the end, our NSF proposal was rejected, and the team decided to edit and resubmit to the same grant after encouragement from a program manager from NSF. However, the second proposal was also rejected. After these rejections the team decided to scale down the project, seek funding from different sources, and apply for a different NSF grant focused on ISE. We found that the biggest critique of our proposals was related to the concept of co-creation with the community, which is embraced by the NSF in conversations and webinars but was not embraced by reviewers. Both proposals proposed using the first year of the grant to co-create and test culturally relevant ISE with community members, but both review panels mentioned that our proposal lacked detail about the ISE we planned to implement. We did provide examples of previously researched ISE that could be adapted during co-creation. Our recommendation for other researchers would be two-fold. First, it is worth the time to identify funding sources that have supported co-creation and/or CBPR in the past, as they likely have a better understanding of this approach and its strengths. Second, if submitting proposals to other, perhaps more general, funding sources, it is important to include a clear and compelling description of why these approaches are important to take in a way that reviewers biased toward more traditional research approaches can buy into. For example, what can they do that other approaches cannot?

#### 7.6.1. Key Outcomes and Recommendations

**Figure 4:**
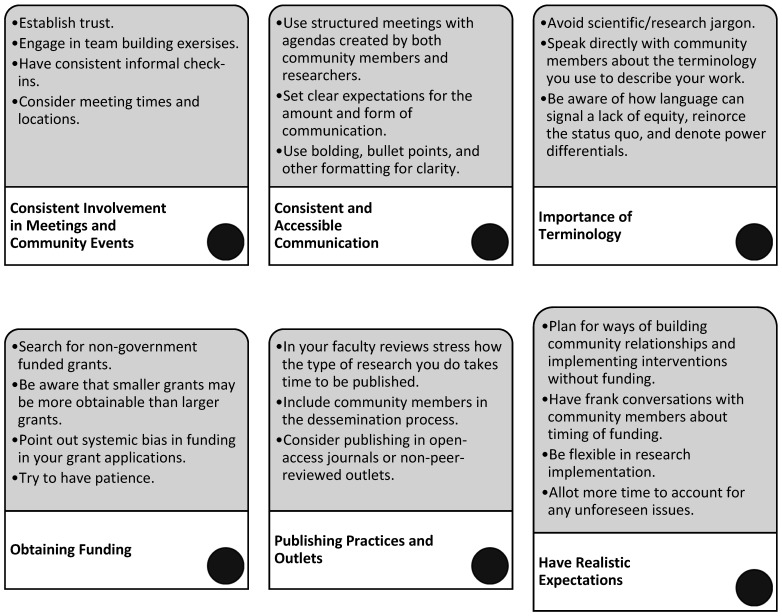
Summary of key outcomes.

After two years of working with our community-team members, we have found several components of our CBPR approach that have been working well that serve as the key outcomes of the present work. In the following paragraphs we describe what has been working well, the challenges we have faced, and our recommendations for other CBPR researchers seeking to form authentic and equal relationships with community members (see Figure 4 for a summary), particularly in communities that are predominantly Black. Be that as it may, we advocate for researchers to consider the unique historical and current experiences of people from both social identity groups. We also encourage researchers to include community members in the decision of which recommendations are appropriate, culturally responsive, and feasible.

#### 7.6.2. Consistent Involvement in Meetings and Community Events

Our regular meetings allowed us to develop a core group of community members and researchers that formed our team. On the researcher side, all the authors on this paper attended nearly all community meetings after they joined the team—save for infrequent pre-planned conference travel and vacations and researchers who left the team. In addition, researchers attended services at two churches and three church events. Church events were a picnic, a Burgers-for-Backpacks event, and a youth fishing event. During these events we had conversations with any non-team community members who wanted to talk about STEM and Flint youth. We had regular community participation in our team meetings as well.

Although we had consistent community-member attendance at team meetings, our hope was that each church would send a representative to attend at least some of the meetings; however, this was not the case. Out of the initial ten churches we formed relationships with, only seven churches sent representatives to the meetings after the first meeting. If we had representatives from more churches, we could be sure that the STEM summer camp and STEM activities we have been co-creating are appropriate for Flint youth across the city rather than in certain churches and their surrounding neighborhoods. More importantly, we would have the opportunity to reach more youth in the city, which could help us increase their interest in STEM and ultimately their decision to pursue STEM. Their decision to pursue STEM is key to reducing minoritization in STEM and providing them with opportunities to earn more money—regardless of whether they attend trade school, a community college, or a university.

One way we can try to overcome this challenge is to work harder to establish trust between research team members and community team members who show interest in participating. Brush et al.,’s (2020) review of CBPR research in which partnerships lasted more than four years found that trust between researchers and community members influenced community partners’ long-term involvement (Caldwell et al., 2015; M. K. Chambers et al., 2015; Hicks et al., 2012; Jagosh et al., 2015). In CBPR, trust can be established and maintained in multiple ways. For example, through consistent and intentional efforts to foster team building, having mutual benefits between partners, and acknowledging what unique skills community members have to offer (Baquet et al., 2013; Hicks et al., 2012; Pivik & Goelman, 2011; Vaughn et al., 2017).

When forming new relationships with potential community team members in the future, we could incorporate team-building exercises into some meetings, have consistent informal check-ins with community team members, and/or make a newsletter that both researchers and community members contribute to. We could also make the mutual benefits clearer, perhaps by coming up with a list of ways we as researchers benefit and inviting community team members to do the same. This could be followed up with a meeting where everyone discusses the benefits to both types of team members and equity is discussed. Importantly, when working in racially diverse teams it is important to talk openly about issues related to race. Psychological research indicates that taking a colorblind approach in which race is ignored can undermine feelings of trust and belonging (Cohen & Garcia, 2008).

Another way to retain interested community members is to rethink the location and format that meetings are held. As previously noted, our meetings were held in the evenings via Zoom, in person at the local library, and in person at two congregations. The CBPR literature indicates that there are strengths and limitations of meeting virtually versus in-person. For example, compared to virtual meetings, in-person meetings allow for interpretation of non-verbal cues and gestures, gives community members more privacy, and eliminates the need for an internet connection (Pocock et al., 2021; Roberts et al., 2021). Moreover, there is some evidence that community members prefer in-person contact (Torres et al., 2020). On the other hand, virtual meetings can create a safe environment for community members where they may be more willing to discuss sensitive issues (Pocock et al., 2021; Valdez & Gubrium, 2020). In addition, virtual meetings can reduce power discrepancies between researchers and community members and alleviate the need for community members to find transportation, childcare, etc. (Pocock et al., 2021; Roberts et al., 2021).

Perhaps scheduling the date, time, *and location* of meetings around both researcher and community team members’ availability before each individual meeting would encourage continued participation. It is feasible that finding new locations closer to some of our partner churches for some in-person meetings could encourage more community members to actively participate and become team members. This could allow people without internet access and/or transportation to attend the meetings. We can also continue to hold both in-person and Zoom meetings but save conversations about sensitive issues for Zoom meetings. The most effective way to figure out how to optimize participation in community meetings is likely to have conversations with people interested in being involved that do not end up attending events. We would also recommend assessing meeting dynamics with community members on a regular basis.

When thinking about potential locations for meetings it is important to discuss psychological research showing that, just as there can be prejudiced people, there can be prejudiced places (Murphy et al., 2013, 2018). Prejudiced places are contexts in which minoritized individuals experience threats to their identity based on features of the environment (Murphy et al., 2013). For example, some physical spaces may feature pictures that only contain White people or room names honoring White individuals. These types of features may be threatening to minoritized individuals and signal the potential for them to be stereotyped or devalued in that setting (Cheryan et al., 2009; Czopp et al., 2015; Steele et al., 2002). Thus, researchers should carefully consider the characteristics of the physical spaces they hold meetings in to ensure that minoritized team members feel safe, valued, and respected.

### 7.7. Consistent and Accessible Communication

Consistent and accessible communication with prospective community team members could also help with sustained community engagement. Work in the CBPR domain highlights the importance of effective communication with community members (Israel et al., 1998; Mayan et al., 2016; Minkler & Wallerstein, 2008). Good communication between researchers and community members maintains trust and is associated with prolonged involvement (Dave et al., 2018). Importantly, communication that is frequent and consistent positively affects relationships with community members and the success of the projects they are working on (Baines & Regan de Bere, 2018; Mikesell et al., 2013; Wine et al., 2017).

Overall, communication between researchers and community members has been both consistent and frequent. Meeting announcements were initially emailed by an administrative assistant that served as a point of contact throughout this endeavor; however, university team members eventually reached out to community members individually as well. An administrative assistant maintains a contact list of attendees, email addresses, and phone numbers along with meeting notes. General messages or announcements related to our team’s work include everyone on the team for transparency, which was supplemented with emails among team members to coordinate event attendance and ask follow-up questions.

Although communication between research and community team members has been good for the most part, we still face challenges that are worth noting. For example, CBPR research highlights the need to listen, evaluate, and respond to information that is shared between team members (Baines & Regan de Bere, 2018). Response rates from research team members’ emails to community team members could sometimes be inconsistent—although some community team members did always respond. We think this could be due to a combination of different issues. For example, when rereading emails between researchers and community team members we found that researchers could have been clearer about expecting a response to the email. Moreover, expectations for contact were never discussed at team meetings. Another similar challenge is communication between community team members and their congregations. Some of our community team members have told their congregations about our partnership and how we are hoping to help the community, but not all have. We think this is a consequence of no formal request to do so being made. Meeting discussions about the benefits of introducing and reminding non-team community members of our partnership could strengthen our team and set us up to request that they participate in our project. Taken together, this suggests that our team meetings could be improved to facilitate better communication.

This leads to another challenge. There were many meetings where a lot of time was spent on discussing the same things covered at other meetings. Although the topics discussed were important and worth returning to, both community team members and researchers think our meetings could be more productive. CBPR research indicates that structured meetings can improve communication (Drainoni et al., 2023). Thus, we would recommend creating an agenda for each meeting with the expectation that we need to get through the agenda (or most of it). In consideration of power dynamics and the need for equality, both researchers and community members should contribute to this agenda. We are taking this approach moving forward.

In addition to using a co-created agenda for meetings, we would recommend that team members are clear about what they need from other team members in both in-person and email discussions. Using bullet points, bolding, and other formatting could make key points of emails clearer. A clear indication of if (and when) a response or resolution is needed could also improve communication. Indeed, establishing guidelines for communication reduces misunderstandings between team members (McShane & Von Glinow, 2003; Simanullang et al., 2024). Moreover, psychological research indicates that people like information that is easy to process (Kahneman, 2013). Depending on team member preferences and access, we could also expand our methods of communication to accommodate different preferences and better reach all team members. Research suggests that offering multiple platforms for communication can improve the transmission of important information (Daft & Lengel, 1986; Peterson & Seligman, 2004).

### 7.8. The Importance of Terminology

Also important is the terminology that we use when communicating. Using the term “study” versus “project” became a point of discussion as the faculty members interacted with community members. Although the faculty were there to offer educational opportunities and inspire change, there had to be research questions attached to secure funding from a federal funding agency. Referring to our work together as a study felt too reminiscent of some previous researchers’ exploitation of Flint residents to help them until their studies were finished. Very open and direct conversations were had to determine how to best manage this conundrum so that equitable relationships were established. Ultimately, we chose to refer to our work together as a project to avoid any negative connotations with the words “study” and “research.”

Another linguistic decision we made as a team was a choice to use the words “listening session” instead of “focus groups” when referring to our plans to talk with parents, caregivers, and other influential adults in Flint youth’s lives. As researchers, our initial approach was to use the term “focus group,” but as we were talking with community members about recruiting people to participate in these focus groups, we realized that “focus group” was an academic term that failed to express our wish to really listen to what community members have to say. It seemed sterile and did not convey that our intentions were to listen to community members to help the community, that is, not just for our personal gain, but for the community too.

Although we tried to be careful in the terminology we used, it could be challenging at times. For example, as researchers we often use the word data and talk about collecting data. We tried to avoid using the term data as it could send the message that we care about the data more than the community. However, because the word data is in our normal lexicon, it was difficult to remember to do so sometimes.

Overall, we recommend that researchers and community members agree to have frank and open conversations with each other about the most appropriate way to describe the work they do together. However, researchers should be wary of putting the onus on community members, particularly when working with people from stigmatized groups. Psychological research indicates that members of stigmatized groups should be able to choose to share their personal experiences because when they are pressured to do so it can be disempowering (Vorauer & Petsnik, 2023). Moreover, putting the onus on stigmatized group members can perpetuate harmful power dynamics, tokenize the experiences of stigmatized individuals, absolves non-stigmatized people’s responsibility to educate themselves, and can promote a sense of entitlement. Instead of putting the burden on stigmatized individuals, non-stigmatized individuals should take initiative to teach themselves about diverse perspectives, systemic racism, and other important concepts like microaggressions. Indeed, psychological research indicates that all of these actions are characteristic of dominant-group allyship according to people of color (K. T. Brown & Ostrove, 2013; Ostrove & Brown, 2018; Park et al., 2022).

### 7.9. Obtaining Funding

Obtaining funding has been, and continues to be, the biggest challenge we have faced. In the last two years we have submitted two NSF applications and three smaller grants (two internal). We received one of the smaller internal grants—which we’ll talk more about in the Discussion—and hope this will increase the chances of our work being funded on a larger scale, starting with a different NSF grant focused on ISE. We are not alone in our struggle; other CBPR researchers also report a lack of funding as a major barrier to success (Amauchi et al., 2022; Lowry & Ford-Paz, 2013; Mirra & Rogers, 2016). In recent systematic review, 61% of social science CBPR projects received funding, compared to 76% of health-related CBPR projects (J. Y. Lee et al., 2024). Out of all 97 projects that received funding across fields, one third reported more than one funding source, and most of the funding was obtained from health-related funding organizations. Taken together, the literature highlights both the lack of social science funding opportunities for CBPR researchers in fields like education and psychology and the lack of CBPR in the social sciences.

One reason for this discrepancy may be that obtaining funding for CBPR can take years (Lowry & Ford-Paz, 2013), which is consistent with our experience. Three researchers are early in their career and have spent a lot of time preparing grant applications. When combined with attending meetings and community events, our early career researchers have performed substantial amount of work, above and beyond requirements for tenure—essentially making some of the hours they spend on the project “unpaid.” While our early career team members are committed to the project and in enduring relationships with community members, they have experienced feelings of devaluation and stress during this project. This is consistent with the experiences of other early career researchers doing CBPR (Fletcher et al., 2022; Lowry & Ford-Paz, 2013). Although our university supports a long-term investment in the community, there is very little internal funding to allow for such an investment—which is common for primarily undergraduate institutions. This is often the case for other CBPR researchers who have also acknowledged that some academic structures incentivize profits for the university (Fleming et al., 2023).

Another reason for this discrepancy and relevant to the racial makeup of both our university and research team, CBPR funding has traditionally gone to historically and predominantly White institutions and faculty members (Fleming et al., 2023). According to the Carnegie Classification of Institutions of Higher Education, R1 universities are more likely to receive large grants (e.g., National Science Foundation, 2024), but there are no R1 historically Black universities. There are only 11 R2 historically Black universities (out of 135), despite there being 104 historically Black universities in the U.S. (The Hundred-Seven, 2024). These trends highlight how systemic racism occurs at multiple levels and in multiple ways—ultimately affecting our ability to help Flint youth and decrease racial minoritization in STEM. Although our university is not historically Black, reviewer comments on our first NSF submission questioned whether our university had the capacity to support our proposed project in a majority-Black city.

These funding issues highlight a vicious cycle of underfunding. Schools are underfunded, which creates racial disparities. Institutions make calls for increased funding of CBPR and ISE to ameliorate these racial disparities, but when it comes time to fund that type of research and support researchers in their efforts no funding is awarded. In other words, underfunding at multiple levels maintains the status quo and continues to leave people in cities like Flint behind. For true change to occur there needs to be both monetary justice (Belfield, 2021) and relational justice (Bennett, 2019) at all levels.

Despite these challenges, our experiences taught us a few things that future researchers should consider. First, we suggest that both research and community team members search for smaller grants as they work toward obtaining the larger grants needed to sustain community involvement. Although this requires teams to scale down their projects, smaller grants can give teams the opportunity to explore specific components of their project that may ultimately lead to a greater probability of success when projects are well funded with larger grants. They can help the team identify and prioritize goals of the project. Second, we suggest that researchers include a discussion of their capacity, the importance of funding research in your community, and, if appropriate, point out systemic bias in funding. Finally, we encourage persistence and a willingness to be flexible. You may not be able to do exactly what you set out to do. It is important to be open to shifting the direction of the research if the community will still benefit and community team members agree.

### 7.10. Publishing Practices and Outlets

This was our first effort to disseminate our work. We appreciate this journal’s willingness to publish a description of early parts of the research process, occurring before methods are finalized and data are collected. Given how much time is needed to build authentic relationships with community members and co-determine the research question and method, dissemination of early experiences such as ours is important; it assists researchers who are thinking about doing, and starting to do, CBPR. Dissemination is also important because disseminating research and using it to inform other research is associated with the success of CBPR projects (Rodriguez Espinosa & Verney, 2021). The publication of CBPR research in the social sciences and STEM is particularly important because there are far fewer published works of research in these fields compared to health-related research, which dominates published CBPR work (J. Y. Lee et al., 2024).

Consistent with early career researchers’ experiences trying to obtain funding, pressure to publish presents a challenge when truly committing to effective and equitable CBPR. Due to its unique and relatively novel approach to research, it can take a long time to get CBPR published (Teixeira & Kennedy, 2022). Balancing tenure demands for publication with investing the time and resources needed to maintain community relationships has been difficult, which other CBPR researchers have mentioned (K. H. Collins, 2018). The “publish or perish” model used in the last two decades is inconsistent with the way that CBPR is conceptualized and performed (Israel et al., 2006). This indicates that both universities and publishers need to be amenable to changing reward structures and publication requirements to be more inclusive of CBPR.

One advantage of CBPR is that it involves community members in the dissemination of their joint efforts (S. E. Collins et al., 2018). This can strengthen the relationship between community members and researchers and provide community team members with contextual information and insights into the research process from researchers’ point of view. Importantly, we recommend that research teams consider alternatives to academic publication when needed. One review of CBPR found that nearly half of the publications that were identified were outside of peer-reviewed journals (S. E. Collins et al., 2018). We have not pursued non-academic publishing, but we intend to in the future.

### 7.11. Have Realistic Expectations

One major take-away from our experiences thus far—and our recommendation for other researchers—is the need to have realistic expectations when planning CBPR projects. As previously mentioned, funding and publication can take a long time to occur. Having realistic plans for ways of building community relationships and implementing interventions without funding is essential. It is also essential to have frank conversations with community members about the timing of funding and realistic plans. Making sure that community members have clear expectations, and an understanding of academia and researcher restrictions, is important in CBPR (Caldwell et al., 2015; S. E. Collins et al., 2018). Upon reflection, we believe we had realistic expectations for building relationships with the community, but we could have been more realistic about our ability to perform research without a major funding source. Our community team members have been very supportive of our efforts to obtain funding, but in hindsight, we could have performed a better job of explaining how long it can take to obtain funding and the constraints that researcher team members face. We plan to have conversations with our community team members to ameliorate this issue.

Realistic expectations are also important to maintain throughout the course of CBPR projects (Sánchez et al., 2021). We learned that we needed to be flexible in research implementation. For example, we wanted to hold listening sessions (i.e., focus groups) with Flint parents to better understand what tools we can offer them to help them support their children’s journey in STEM, but in one of our monthly meetings our conversations with community team members we realized that interviews with parents at church events was a better place to start. Now, nearly a year later, we are returning to listening sessions with updated discussion prompts based on our interviews with parents. Related to flexibility, we have also learned that we need to allot more time to account for any unforeseen issues that may arise (Black & Faustin, 2022). For example, we originally expected that recruitment of Flint youth would be relatively easy given how many churches we were working with; however, the lack of active participation by some churches suggests that we should adjust our expectations. We recommend that other researchers be flexible at all stages of the CBPR process.

## 8. Discussion and Conclusions

Overall, our CBPR experiences thus far have been positive and informative. We are optimistic about our team’s ability to start to address minoritization in STEM. As previously mentioned, Flint is a unique, predominately Black, city that is burdened by systemic racism in the education system, disinvestment, urban decay, and a distrust of the government and researchers (Carrera et al., 2019; Hightower, 2015). Moreover, Flint schools are not adequately preparing Flint youth to pursue STEM, despite Flint’s automotive history. Thus, a major goal of our project is to co-create ISEs for Flint youth outside the formal education system. By using a CBPR model, we have been able to form authentic relationships with community members and have started to develop ISEs that are culturally relevant, responsive, and asset-based.

Although we had hoped to be working with Flint youth already, our journey thus far has provided us with invaluable lessons that we hope will help other CBPR researchers. We learned about the need for a diverse research team and the importance of social-networking within the community to form relationships with community members that lead them to join the research team. We also learned to have more realistic expectations. Our original goals were to run a STEM summer camp using the vacation Bible school model and create STEM learning activities that could be hosted at different churches on a frequent basis. We later found out that many of the churches have stopped having the Bible summer camps since COVID, which impeded our progress. Because of this, we have not accomplished this goal, but we have refined our ideas, started to co-create ISE, and have laid the foundation for our ability to reach these goals in the future.

A review of our experiences thus far led to six primary takeaways.

First, we learned about the importance of consistent involvement by both research and community team members. We recommend that research teams engage in team building exercises, consider the most equitable timing and location for team meetings, and continuously work on developing and maintaining trust.

Second, we had favorable experiences with consistent and frequent communication, but we can see room for improvement and suggest the use of things like structured meetings and newsletters to improve communication.

Third, our work with community members led to careful considerations about what terminology we used to avoid making the community feel like “lab rats.” We recommend that research teams challenge themselves to be cognizant of the language they use and have frank and open conversations about what language is respectful and mindful of power dynamics.

Fourth, we learned that obtaining funding was going to take much longer than anticipated, resulting in a need to apply for smaller grants and adjust both research and community team members’ expectations.

Fifth, we had to similarly adjust our expectations for the publication of our research and suggest that universities and journals adjust their publication requirements—like this journal has—to publish more CBPR at various stages of development and implementation. Related to both obtaining funding and publishing, we noted the challenges for early career researchers engaging in CBPR. Addressing these challenges is difficult given that researchers have little control over tenure requirements and the mindset of academia. Thus, we recommend that early career CBPR researchers go into CBPR projects with an understanding that they will likely be committing to “unpaid” hours of work and find ways of managing the stress associated with the demands of CBPR research.

Sixth, and a theme across our major takeaways, we learned about the need to have realistic expectations about every part of the research process and to remain flexible at every stage of the research process.

Our review of our experiences thus far makes several novel contributions to the literature. First, our review contributes to the limited research on CBPR in STEM among minoritized individuals. Unlike past CBPR STEM research (Calabrese Barton & Tan, 2018; Kennedy et al., 2016; Nation & Hansen, 2021), our work provides an overview of a CBPR experience where *community members* are treated as equal partners. Given the length of our relationship with the community we offer novel insights into developing successful partnerships in a city like Flint. We also offer novel insight into CBPR STEM research by reviewing experiences that are not linked to formal advocacy groups, who often have more resources than groups like ours. Finally, our work examines a CBPR project from both an educational and psychological perspective.

As previously stated, a systematic review by (Rodriguez Espinosa & Verney, 2021) revealed only 7 (out of 311) published CBPR-psychology articles related to education and curricular development. The current work adds one more to this list. In addition, unlike previous work, the current work provides an example of psychological research in which co-researchers are community members rather than undergraduate or high school students (e.g., Guy & Feldman, 2023; Jacquez et al., 2020; Siciliano et al., 2018). Our work also adds to the literature on CBPR methods in psychology by reviewing CBPR based on a long-term relationship with community members.

Finally, and most importantly, the present work reviews CBPR at the intersection of psychology and STEM, which is the only work we know of to date to do so aside from Navickis-Brasch et al. (2014), which is a published conference paper. The successes and challenges that Navickis-Brasch et al. (2014) faced provides a nice way of highlighting the current work’s contribution. Navickis-Brasch et al. (2014) reported that community members did not feel like equal partners, there was a lack of communication, and communication could have been clearer. In our work we built an equitable relationship with community members, actively communicated with community members, and worked to have clear communication. Navickis-Brasch et al. (2014) also reported a lack of co-development and community concerns about prioritizing publication over the needs of the community. We feel that our work provides an example of successfully avoiding these problems. Importantly, we still need to improve in all these areas, and we recognize the importance of committing to frequent evaluations, a willingness to change, and community responsiveness.

### 8.1. Broader Impacts

Overall, our review of our experiences has multiple broader impacts. It is our hope that our experiences will help other CBPR researchers to successfully build and maintain equitable and power-conscious relationships in their communities. We provide insights into sustaining long-term involvement, communication dynamics, funding, and publishing—all of which can help CBPR researchers to have more realistic expectations and avoid overpromising. We are also straightforward about the level of commitment required and how this has a differential impact on early career researchers. By better preparing CBPR researchers, particularly those in cities like Flint, there is a greater chance that CBPR projects will be successful and make a meaningful impact on the communities they serve.

Another broader impact of our work is the development of a more trusting relationship between the Flint community and the university. Although one CBPR project is certainly not enough to overcome years of mistreatment by the government and researchers, it is enough to start to move the needle. Located in downtown Flint, the university has been a central part of the city of Flint for years (Hightower, 2015). Our team’s inclusion of the Urban Institute and the Office of Research demonstrates support from the university in pursuing our work. It is our hope that our relationship with the community continues for many years and fosters an interest among university researchers to use their skill sets to help the community.

### 8.2. Limitations

Despite the clear contributions of our work and its broader impacts, it is important to acknowledge the limitations of our review. To start, our work was not all inclusive. We wrote about six aspects of our experiences, but due to space constraints, we did not get to cover other important parts of our experiences such as information about our development of ISE and interpersonal challenges. We also did not touch on negotiating and addressing conflict, which is an important part of CBPR (Baines & Regan de Bere, 2018). Another limitation is that we did not include educators, school administrators, or educational researchers on our team. Indeed, this was brought up on our reviews of one of the NSF grant applications. We believe that our focus on co-creation with community members and working outside the formal education system—which is flawed in the city of Flint—makes the need for the involvement of such personnel essential. Moreover, adding another researcher to the team requires paying an additional researcher out of grant funds, reducing the amount of funding used to help the community.

### 8.3. Future Directions

In addition to addressing the challenges we reviewed above, moving forward we have a myriad of plans to strengthen our relationship with the community and move our research agenda forward. First, we intend to have a better assessment of relationship between research and community team members. Thus far our assessment has been limited to our own observations and reviews of past meetings. However, in line with other CBPR (for a review see Brush et al., 2020), we intend to administer informal surveys to our community team members. We also plan to have a meeting focused on evaluating our relationship and progress and ways that we as a team can function better. Second, we intend to attend more community events that are more social in nature, rather than research-focused. Third, we plan to apply for smaller grants in addition to large grants so that we can start actually working with Flint youth. Finally, given grant reviewer comments, we plan to pare down the research we propose in our grant applications to focus on one component of our research agenda at a time. For example, we intend to apply for funding for our summer camp and our ISE nights in separate applications.

Related to this idea, the one small grant we have been able to obtain specifically focuses on Flint parents. When we talked to Flint parents at the events we attended, we found that most of them had no idea where to start and what to do to help encourage their children to pursue STEM. They all wanted to support their kids; they just did not know how. Thus, we realized that, to make our work with Flint youth work, we also need to target their parents. Our funding will allow us to have listening sessions with parents and formally survey parents to help us design an information night where they can get all their questions answered.

Notably, many factors influence the participation of students of racial minorities in STEM fields, but chief among them are the influence of parents and the impact of mentors or role models (Kricorian et al., 2020; Nguyen & Riegle-Crumb, 2021). Family encouragement and support are widely recognized as essential starting points for students who attain educational success in STEM fields (Burt & Johnson, 2018; Crisp & Nora, 2012). Parental involvement and engagement have been found to have a key influence on students’ identification with scientific thinking and problem solving (Mishra, 2012). Being immersed in a family and social environment where talk of science is routine and exposure to science media in childhood helps to predict STEM identity in higher education (Dou et al., 2019). Hall et al. (2011) examined those factors exerting the most influence on high school students’ career choices and identified the top four factors as personal interest, parents, earning potential, and teachers, in that order. The same study observed that students required career knowledge from parents and teachers to develop their interest—in effect increasing the influence of parents and teachers shapes students’ careers. These results are consistent with other studies (e.g., Burušić et al., 2021; Falk & Meier, 2021; Maltese & Cooper, 2017; Mau & Li, 2018; Razali et al., 2018).

Finally, it is important for future researchers to further incorporate psychological tools and knowledge into their CBPR methodology. By leveraging psychological research on people’s motivations, perceptions, cognitions, and behavior, scientist can build and strengthen their relationships with the community in multiple ways. Scientists can also leverage psychological research to inform their research objectives and the way they think about the impact of their research.

## 9. Conclusions

Cities like Pittsburg, Baltimore, Cleveland, Detroit, and Flint were built on and continue to thrive because of innovations in STEM. However, historical and contemporary systemic racism consistently impedes the ability of youth from these cities to achieve the level of education they need to successfully pursue STEM careers. Youth in predominantly Black cities, like Baltimore, Detroit, and Flint, have unique life experiences that need to be considered in researchers’ efforts to reduce racial minoritization in STEM. A one-size-fits-all approach fails to acknowledge youths’ lived experiences, the importance of cultural relevance, and features of the context that may make certain types of interventions ineffective. To acknowledge these factors, a CBPR approach can be, and arguably should be, used. To effectively and ethically help people in cities like Flint—both within and outside the domain of STEM—researchers should work with community members to develop their research questions and the best way to answer those research questions in those cities.

## Figures and Tables

**Table 1:** City of Flint school district 2023–2024 state test score proficiency by grade and race.

				MSTEP Math		MSTEP Science		PSAT Math	
Grade	Number of Students	Number of Black Students	Number of Economically Disadvantaged Black Students	Percentage of All Students Not Proficient	Percentage of Black Students Not Proficient	Percentage of All Students Not Proficient	Percentage of Black Students Not Proficient	Percentage of All Students Not Proficient	Percentage of Black Students Not Proficient
3rd	222	148	142	81.10%	83.11%	-	-	-	-
4th	263	172	163	77.57%	84.88%	-	-	-	-
5th	264	175	165	82.20%	86.86%	62.50%	69.14%	-	-
6th	202	151	140	80.20%	83.44%	-	-	-	-
7th	133	103	96	96.24%	84.68%	-	-	-	-
8th	172	126	119	-	-	71.51%	80.16%	88.95%	93.65%
11th	163	133	115	-	-	53.99%	57.14%	-	-

Note. Data provided by the Center for Educational Performance and Information (MCEPI, 2024a, 2024c). Number of students includes students of all races/ethnicities and economic background. Students are economically disadvantaged if they are eligible for free or reduced-price meals.

## Data Availability

No formal data were used for this project.
